# Tbx5 overexpression in embryoid bodies increases TAK1 expression but does not enhance the differentiation of sinoatrial node cardiomyocytes

**DOI:** 10.1242/bio.059881

**Published:** 2023-06-05

**Authors:** Yunkai Dai, Fatemeh Nasehi, Charles D. Winchester, Ann C. Foley

**Affiliations:** Clemson University, Department of Bioengineering, 68 President Street, Charleston, SC 29425, USA

**Keywords:** Sinoatrial node, SAN, Tbx5, TAK1, Embryoid body

## Abstract

Genetic studies place Tbx5 at the apex of the sinoatrial node (SAN) transcriptional program. To understand its role in SAN differentiation, clonal embryonic stem (ES) cell lines were made that conditionally overexpress Tbx5, Tbx3, Tbx18, Shox2, Islet-1, and MAP3k7/TAK1. Cardiac cells differentiated using embryoid bodies (EBs). EBs overexpressing Tbx5, Islet1, and TAK1 beat faster than cardiac cells differentiated from control ES cell lines, suggesting possible roles in SAN differentiation. Tbx5 overexpressing EBs showed increased expression of TAK1, but cardiomyocytes did not differentiate as SAN cells. EBs showed no change in the expression of the SAN transcription factors Shox2 and Islet1 and decreased expression of the SAN channel protein HCN4. EBs constitutively overexpressing TAK1 direct cardiac differentiation to the SAN fate but have reduced phosphorylation of its targets, p38 and Jnk. This opens the possibility that blocking the phosphorylation of TAK1 targets may have the same impact as forced overexpression. To test this, we treated EBs with 5z-7-Oxozeanol (OXO), an inhibitor of TAK1 phosphorylation. Like TAK1 overexpressing cardiac cells, cardiomyocytes differentiated in the presence of OXO beat faster and showed increased expression of SAN genes (Shox2, HCN4, and Islet1). This suggests that activation of the SAN transcriptional network can be accomplished by blocking the phosphorylation of TAK1.

## Teaser

**Summary:** The sinoatrial node transcriptional program is activated by blocking phosphorylation of TAK1 during cardiac differentiation of pluripotent cells, providing a simplified protocol for the in vitro differentiation of cardiac pacemakers.

## INTRODUCTION

The sinoatrial node (SAN) is an anatomically discrete region of specialized myocardium that initiates and propagates electrical impulses in the heart. The transcription factor Tbx5 has been a primary target for studies of SAN differentiation since it was first linked to Holt-Oram syndrome, a disease characterized by cardiac conduction system abnormalities (Basson et al., 1994; Holt and Oram, 1960; Newbury-Ecob et al., 1996).

Tbx5 is a T-box of transcription factor (Basson et al., 1997; Li et al., 1997) that plays a critical role in the specification and maintenance of the conduction system by regulating the conduction system transcriptome (Arnolds et al., 2012; Moskowitz et al., 2007, 2004). Germline deletion of Tbx5 in mice leads to embryonic lethality by E10.5 and severely impaired SAN differentiation (Bruneau et al., 2001). The atrioventricular conduction system fails to mature in Tbx5 haploinsufficient (Tbx5^−/+^) mice, resulting in a prolonged PQ interval (Moskowitz et al., 2004). Together these data indicate that Tbx5 is necessary for the differentiation of the conduction system generally and SAN differentiation specifically.

A transcriptional program for SAN differentiation has been defined through genetic studies, placing Tbx5 at the apex (Christoffels et al., 2010). Overexpression of transcription factors downstream of Tbx5 in pluripotent stem cells has been shown to activate the differentiation of cardiomyocytes with some or all of the characteristics of SAN cells (Ionta et al., 2015; Jung et al., 2014; Kapoor et al., 2013). The role of Tbx5 in these studies has remained to be determined. In addition, these data and ours presented below suggest that induction of specific cardiac fates by transcription factor overexpression may be highly dose-dependent. The differentiation of SAN-like cells from pluripotent cells can also be activated by manipulating intracellular signaling cascades mediated by BMP (Protze et al., 2017) and MAP kinase signaling (Brown et al., 2017; Wiese et al., 2011). Precisely how these signaling cascades impact SAN development is unknown. Induction of SAN-like cells with BMP requires precise temporal control of the treatment regimen, whereas constitutive overexpression of MAP3k7/TAK1 was sufficient to induce SAN phenotypes. However, constitutive TAK1 still exerted control of gene expression temporally in embryoid bodies (EBs), with Tbx5 being upregulating and Nkx2.5 downregulated early in EB differentiation and other SAN genes impacted at later time points (Brown et al., 2017). To test the importance of Tbx5, we developed constructs to overexpress Tbx5 and other members of the SAN transcriptional program under the control of doxycycline (DOX) inducible promoters.

Tbx5 overexpression did cause cardiomyocytes derived from EBs to beat faster but did not activate other signs of SAN differentiation; however, these EBs did surprisingly overexpress TAK1. Constitutive overexpression of TAK1 directed cardiomyocyte differentiation to the SAN fate but also repressed the phosphorylation of TAK1 downstream targets, including p38 and Jnk (Hunter et al., 2019). Since the constitutive overexpression of TAK1 suppresses the phosphorylation of known downstream targets, blocking TAK1 phosphorylation might have the same effect as forced overexpression. To test this EBs were treated with 5z-7-oxozeanol (OXO), an inhibitor of TAK1 phosphorylation. Cardiac cells differentiated in the presence of OXO beat faster, showed morphological changes, and expressed both the SAN channel protein HCN4 and key SAN transcription factors Shox2 and Islet1, supporting this hypothesis. These data suggest that Tbx5 is insufficient to induce SAN fates but also suggest that blocking phosphorylation targets downstream of TAK1 may play a critical role in determining SAN fate. These data provide new insights into the role of intracellular signaling in specifying specific cardiac physiologies. They may also provide a simplified protocol for the differentiation of SAN cardiomyocytes from pluripotent cells.

## RESULTS

**Fig. 1.**
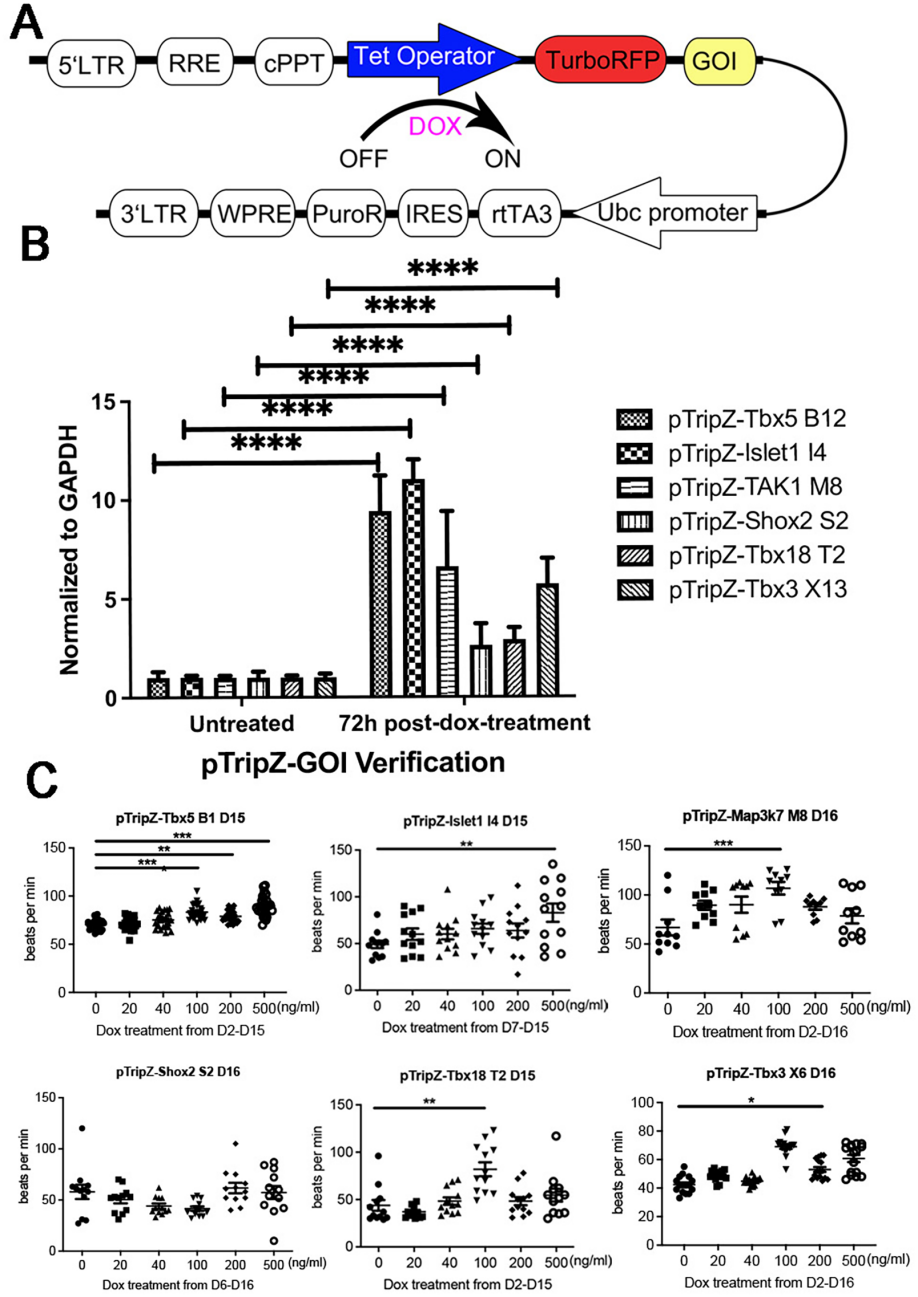
**Clonal Isolation and testing of ES cell lines that conditionally overexpress important SAN genes.** (A) Schematic illustration of the inducible overexpression vector. (B) QRT-PCR data of Tbx5, Islet1, TAK1, Shox2, Tbx18, and Tbx3, 72 h post-DOX treatment compared to untreated cells. Data represent means±s.e. of three independent experiments. Statistical significance was determined by unpaired, two-tailed *t*-test. (C) Manual beat data counting of Tbx5, Islet1, TAK1, Shox2, Tbx18 and Tbx3 overexpressing EBs at day 15 with DOX doses of 0 ng/ml, 20 ng/ml, 40 ng/ml, 100 ng/ml, 200 ng/ml and 500 ng/ml. Statistical significance was determine by ANOVA. *P<0.05, **P<0.01, ***P<0.001, ****P<0.0001.

Mouse embryonic stem (ES) cell lines that conditionally overexpress Tbx5, Islet1, TAK1, Shox2, Tbx18, or Tbx3 were generated using a lentivirus that drives gene expression and a fluorescent reporter under the control of a DOX-inducible promoter. This vector also has a selection cassette with puromycin resistance driven by the ubiquitin promoter TRE::turboRFP-GOI; UBC::rtTA3-IRES-PURO (Fig. 1A). Several clonal lines were isolated for each vector and expression of target genes was confirmed in clonal ES cell lines after 72 h of DOX treatment by qRT-PCR (Fig. 1B).

As an initial test of each cell line's potential to differentiate into SAN cardiomyocytes, each mES cell line was differentiated as EBs and treated with increasing doses of DOX (0-500 ng/ml) (Fig. 1C). In most cases, treatment began on day 2, however, in two instances, Shox2 and Islet1, DOX treatment was started on days 6 and 7, respectively, based on their expression in wild-type ES cells (Brown et al., 2017), since earlier DOX treatment in these lines inhibited cardiac differentiation. The resulting EBs were evaluated for beat rate, an early indicator of SAN differentiation on day 15 or 16. Beating areas were identified based on visual inspection and confirmed as cardiac by expression of GFP from the αMHC::GFP promoter-reporter, which we previously confirmed as a reliable readout of cardiac differentiation from mouse ES cells (Brown et al., 2010).

Tbx5-overexpressing EBs had increased beat rate at 100 ng/ml, 200 ng/ml, and 500 ng/ml DOX treatment. Interestingly although all doses above 100 ng/ml showed increased rates of beating, above this threshold, beat rate was not related to DOX dose. Among the other lines, Islet1 overexpressing EBs showed a modest increase only at the highest dose tested, 500 ng/ml. Tbx3, Tbx18, and MAP3k7 overexpressing EBs showed maximal beat rate at 100 ng/ml but decreasing beat rates at higher doses. Shox2-overexpressing EBs showed no change in beat rate across different doses.

**Fig. 2.**
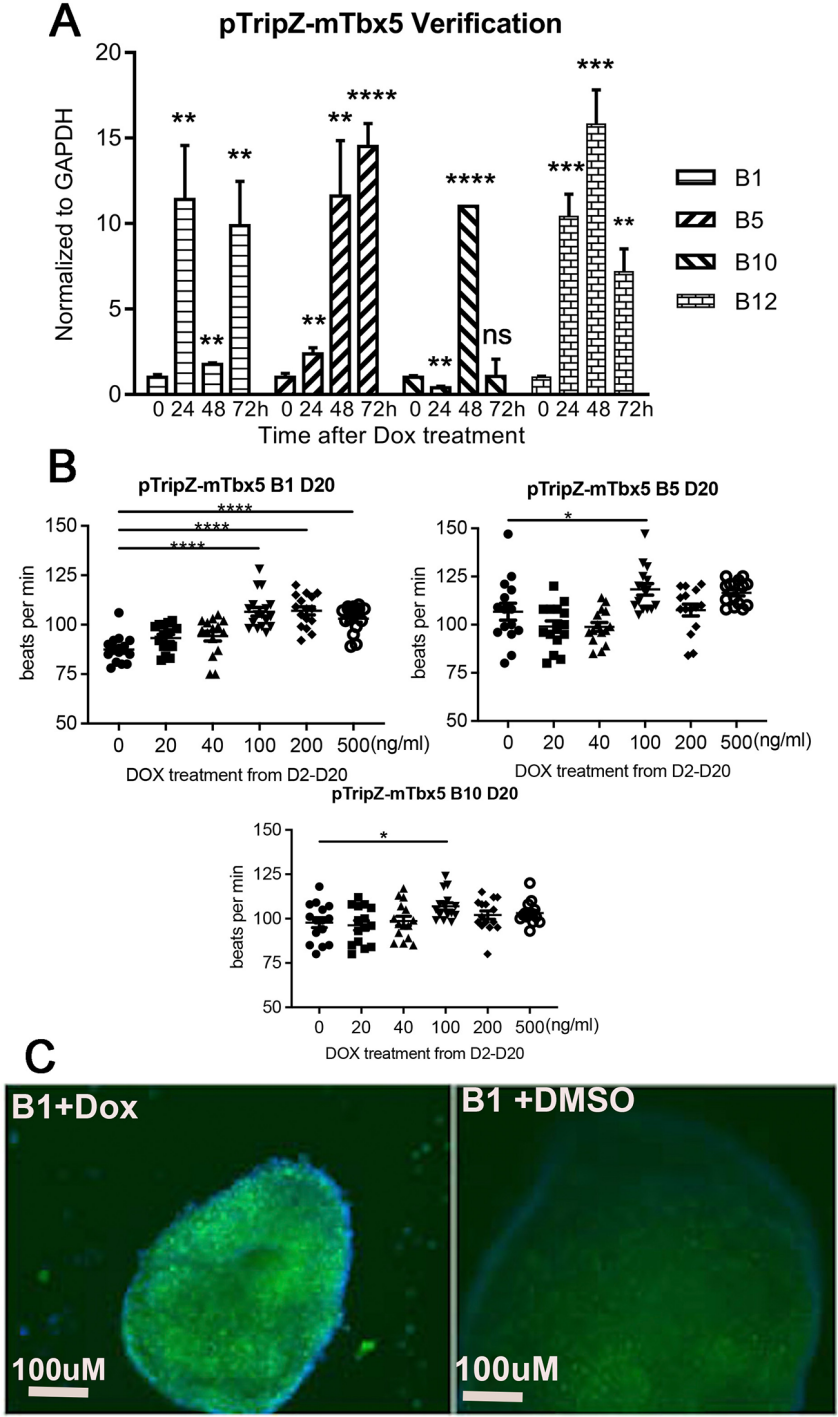
**Verification of pTripZ-mTbx5; αMHC::GFP mouse ES cell lines.** (A) B1, B5, B10, B12 mES cells were treated with 1 µg /mL DOX and qRT-PCR was performed to detect relative expression of Tbx5 at 0, 24, 48 and 72 h. Statistical significance was determined by unpaired two-tailed *t*-test. (B) Manual beat data counting of B1, B5 and B10 EBs at day 20 with DOX doses of 0 ng/ml, 20 ng/ml, 40 ng/ml, 100 ng/ml, 200 ng/ml and 500 ng/ml. Statistical significance was determined by ANOVA. (C) Immunocytochemistry using anti-Tbx5 antibody in B1 mES cells 48 h after DOX or DMSO addition. *P<0.05, **P<0.01, ***P<0.001, ****P<0.0001.

Four independent clonal cell lines overexpressing Tbx5 were tested (B1, B5, B10, and B12). All showed stable, inducible upregulation of Tbx5 transcripts in ES cells, as confirmed by qRT-PCR at 24, 48 and 72 h after addition of DOX (Fig. 2A). Three of the four cell lines showed increased beat rate in 21-day-old cardiomyocytes at one or more dosages of DOX (Fig. 2B). The fourth clonal line showed only poor lineage differentiation and was excluded from further analysis. B1 cells showed the most robust cardiac differentiation of the remaining clones and were therefore used for all subsequent studies. Upregulation of TBX5 protein in response to DOX was confirmed in the B1 line by immunocytochemistry (Fig. 2C).

### B1 ES cells show increased cardiac differentiation unrelated to the overexpression of Tbx5

**Fig. 3.**
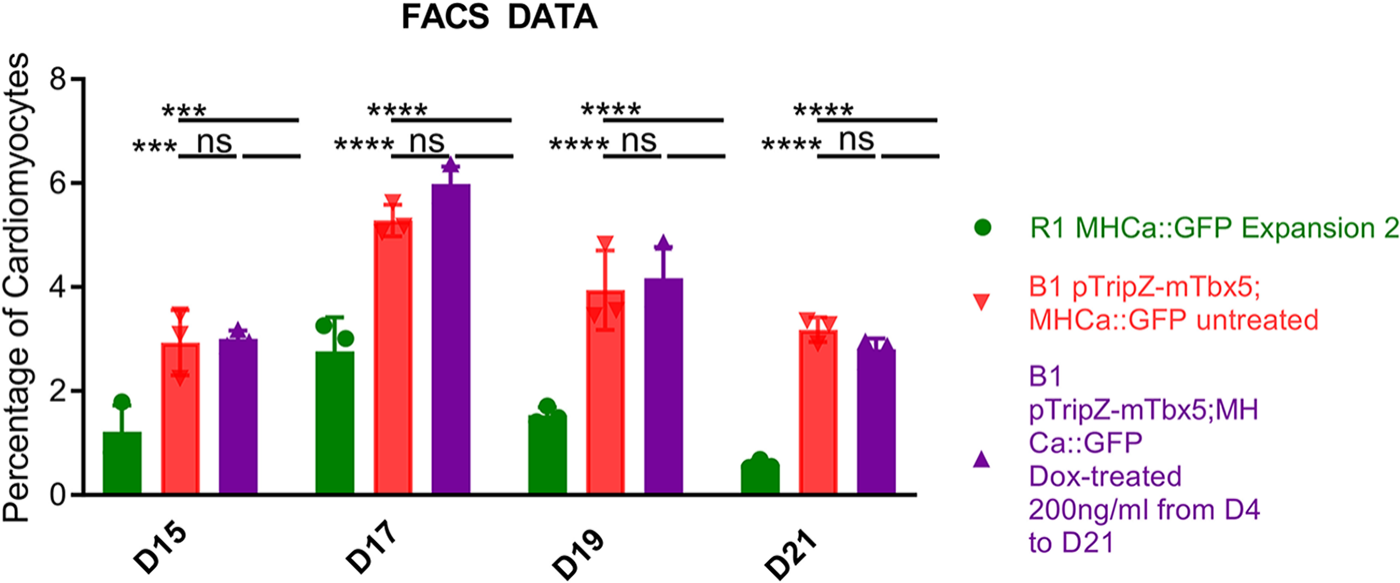
**Cardiac differentiation analyzed by flow cytometry.** The percentage of cardiomyocytes at day 21 of EB differentiation based on the number of cells expressing the aMHC::GFP reporter as a percentage of total cells by flow cytometry. Data represent means±s.e. of three independent experiments. Statistical significance was determined by unpaired, two-tailed *t*-test. ***P<0.001, ****P<0.0001.

Our previous experience studying cardiogenesis using ES cells suggests that clonal isolation can impact the cardiogenic potential of cells. To test this, EBs were differentiated with or without the addition of DOX from day 4 to day 21 (Fig. 3). Cells were assessed on days 15, 17, 19, and 21 by flow cytometry for cardiac differentiation. Cardiomyocytes were identified based on the expression of GFP from the αMHC::GFP transgene and cardiac differentiation expressed as the percentage of GFP-expressing cells. The B1 line, with or without the addition of DOX, showed increased cardiac differentiation compared to the parent cell line (R1). These data suggest that the increase in cardiac differentiation in B1 EBs is due to clonal differences but was not impacted by Tbx5 overexpression.

### Day-21 EBs do not show enhanced expression of SAN markers

**Fig. 4.**
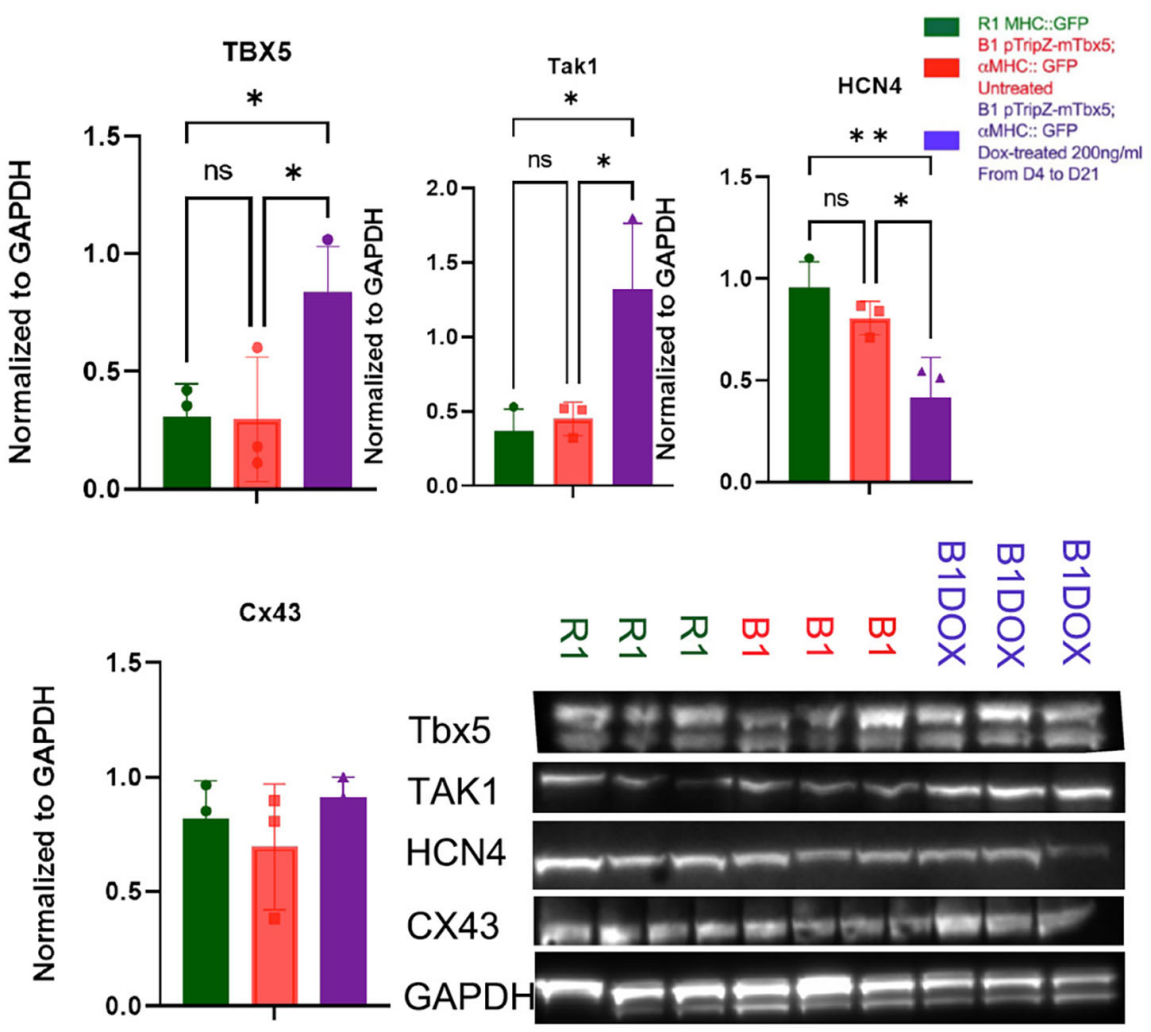
**Relative protein expressions of R1, B1, B1 DOX-treated EBs at day 21.** Tbx5, MAP3k7/TAK1, HCN4 and Cx43 proteins were assessed by western blot. Data represent means±s.e. of three independent experiments, normalized to GAPDH. Statistical significance was determined by unpaired two-tailed *t*-test. *P<0.05, **P<0.01.

At 21 days, DOX-treated EBs continued to overexpress Tbx5, as assessed by western blot (Fig. 4). They also overexpressed TAK1, which we previously identified as a protein that could direct myocardial differentiation in EBs to the SAN fate when overexpressed. Interestingly, DOX treated EBs had decreased expression of the SAN marker HCN4. Protein expression of Cx43, which is expressed in all cardiac cells except the SAN, was unchanged.

### Day-21 cardiomyocytes do not have enhanced expression of SAN markers

**Fig. 5.**
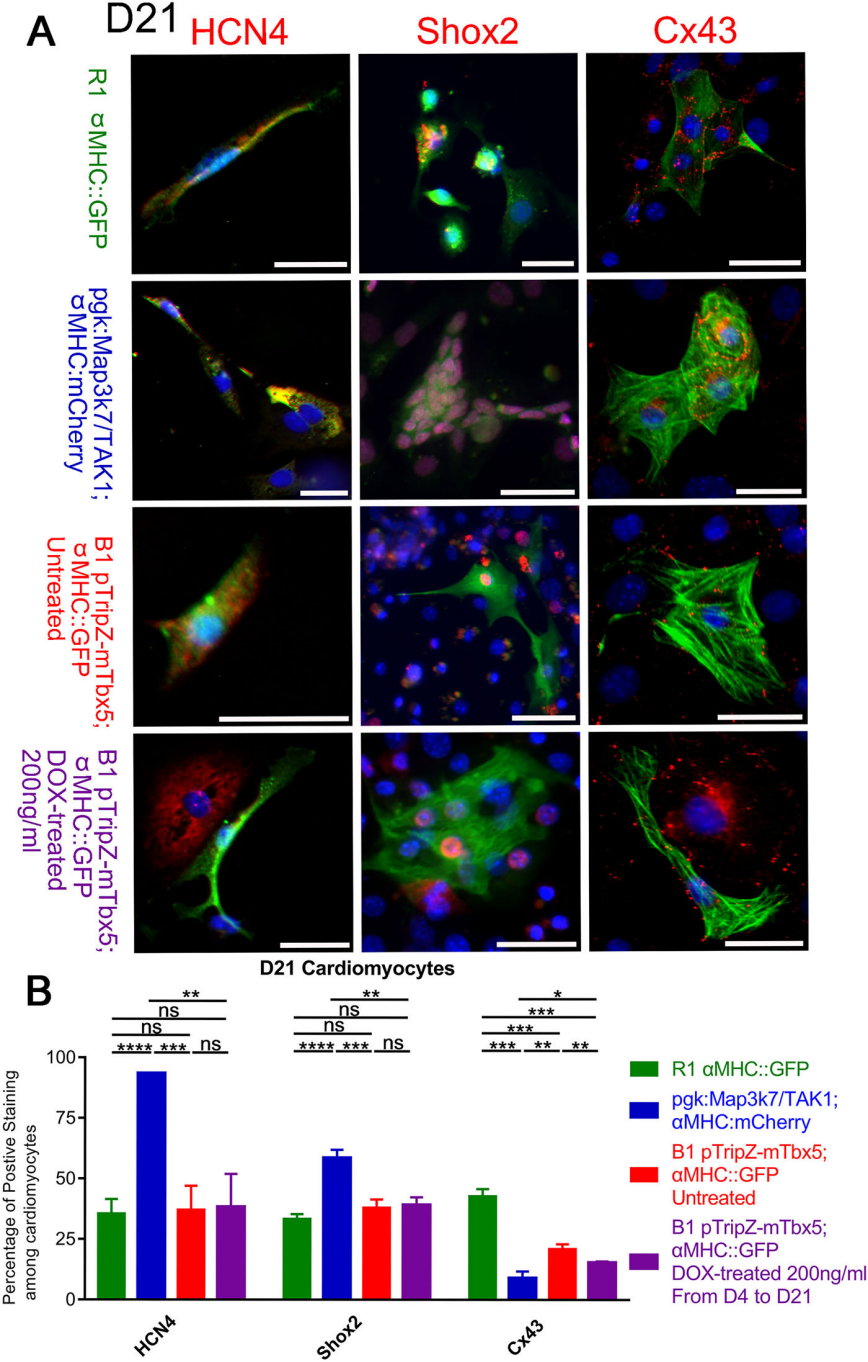
**SAN marker expression in D21 cardiomyocytes.** (A) Representative image of ICC staining of day 21 cardiomyocytes of R1, MAP3k7/TAK1-overexpressing, B1, and B1 DOX-treated EBs. EBs were dissociated into single-cell suspension and replated onto chamber slides. Then these cells were stained with GFP, DsRed or CT3 antibody to identified cardiomyocytes. HCN4 (R1 *N*=124, TAK1 *N*=233, B1 *N*=125, BIDOX *N*=110), Shox2 (R1 *N*=128, TAK1 *N*=175 B1 *N*=116, BIDOX *N*=146), and Cx43 (R1 *N*=108, TAK1 *N*=61, B1 *N*=88, BIDOX *N*=70) antibody were immunostained with red fluorophore, DAPI labelled nuclei blue. Scale bars: 30 µm. (B) The percentage of HCN4, Shox2 and Cx43 positive cells. Data represent means±s.e. of at least three independent experiments. Statistical significance was determined by unpaired two-tailed *t*-test. *P<0.05, **P<0.01, ***P<0.001, ****P<0.0001.

Since cardiac cells represent only a fraction of the cells that differentiated in these EBs, we hypothesized that analysis of single cardiac cells might be required to see changes in SAN markers. To assess the expression of SAN markers, specifically in cardiomyocytes, EBs were dissociated, and individual cells replated in slide wells. Cardiac cells were identified using immunocytochemistry to label cells expressing the cardiac promoter reporters αMHC::GFP or αMHC::mCherry (anti-GFP, or anti-mCherry antibodies). In some cases, cardiac cells were identified by immunocytochemistry using the anti-cardiac troponin antibody (anti-CT3). Individual cardiomyocytes were also immunostained for HCN4, Shox2, or Connexin 43 (Cx43) (Fig. 5A). HCN4 and Shox2 are expressed in SAN cells, and Cx43 is expressed in all cardiac cells except the SAN.

For these studies, we used B1 cells with or without the addition of DOX to test the impact of Tbx5 overexpression. For positive controls, we used the MAP3k7/TAK1-overexpressing cell line PGK::MAP3k7/TAK1; αMHC::mCherry, which directs cardiac cell differentiation to the SAN lineage (Brown et al., 2017). We used the parent cell line R1;αMHC::GFP as a negative control for SAN differentiation. As expected, a higher percentage of cardiac cells derived from the TAK1 EBs expressed HCN4 and Shox2, whereas a lower percentage of these cells expressed Cx43, compared to the R1 parent cell line (Fig. 5B).

Neither the B1 nor the B1 DOX-treated EBs showed increased expression of HCN4 or Shox2, suggesting that Tbx5 overexpression itself cannot activate the SAN transcriptional program. These cells did show decreased expression of Cx43, like the TAK1 EBs (Fig. 5B), but this did not depend on the addition of DOX, so it cannot be related to Tbx5 expression levels. Together these data show that Tbx5 overexpression does not direct cardiac differentiation to the SAN fate.

### Blocking TAK1 phosphorylation activates SAN markers in cardiac differentiation

**Fig. 6.**
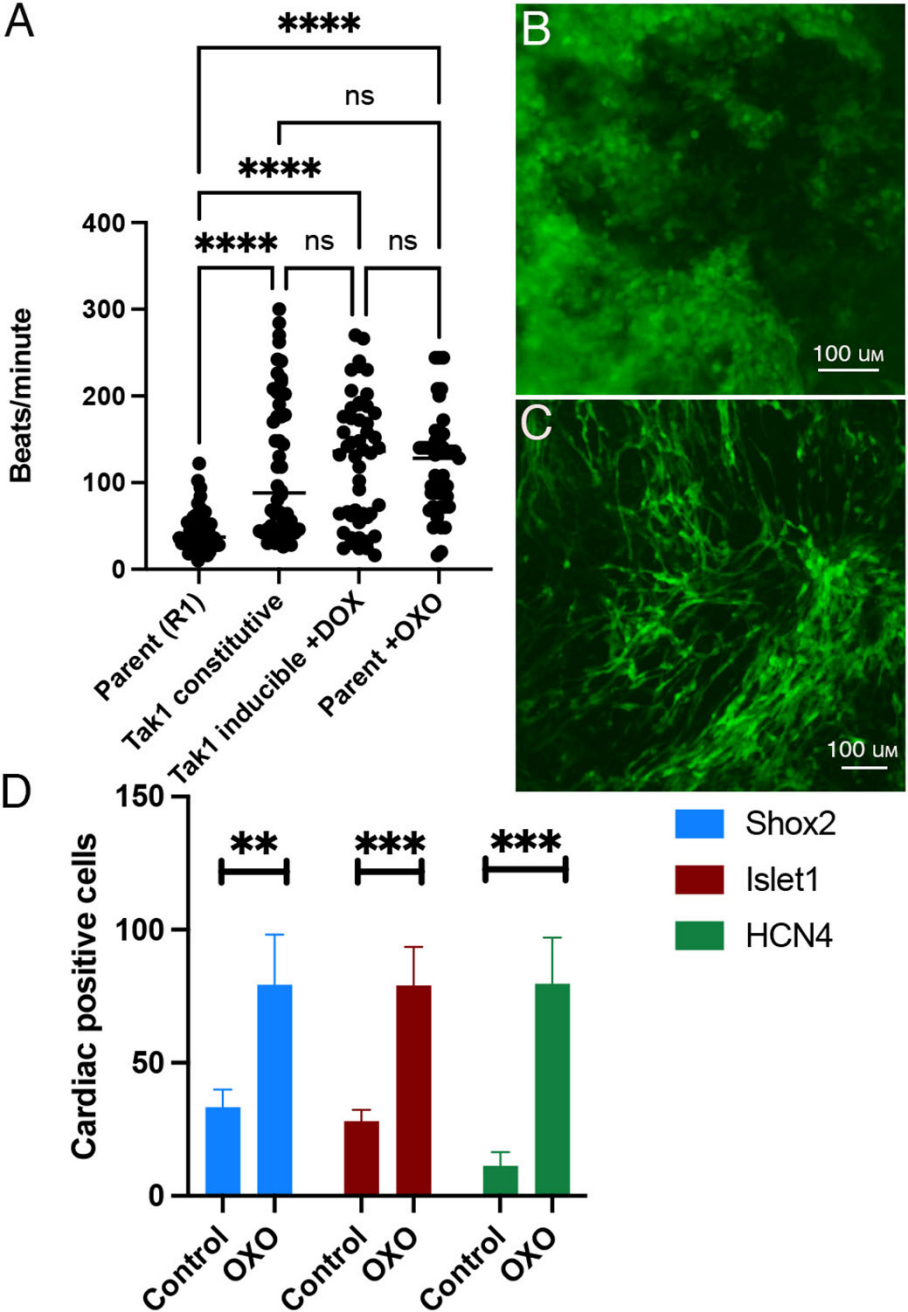
**Blocking TAK1 phosphorylation increases expression of SAN transcription factors in cardiomyocytes.** (A) Beat rate data day 21 cardiomyocytes from R1, TAK1 constitutive, TAK1-inducible with DOX, and R1 with OXO. Statistical significance was determined by ANOVA. (B) Live cell imaging of day 21 cardiomyocytes from R1 and (C) R1 with OXO treatment, imaging fluorescence from the aMHC:GFP cardiac promoter reporter. (D) Quantification of Shox2 (control *N*=110, OXO *N*=80), Islet1 (control *N*=48, OXO *N*=44), and HCN4 (control *N*=118, OXO *N*=123) expression in cardiac cells as assessed by ICC, with and without OXO treatment. Data represents means±s.e. of at least three independent experiments. Statistical significance was determined by an unpaired two-tailed *t*-test. **P<0.01, ***P<0.001, ****P<0.0001.

Forced overexpression of TAK1 using the strong mammalian promoter PGK directed most cardiac differentiation to the SAN fate (Brown et al., 2017). Here however, physiological increases in TAK1 that result from TBX5 overexpression did not induce SAN differentiation. This could be due to a dosage effect, but this is unlikely since the increased beat rate in response to Tbx5 overexpression began at a specific dose of DOX and was not further improved by increasing the dosage (Fig. 1). However, we had previously observed that PGK::TAK1 EBs actually downregulated the phosphorylation of known TAK1 targets (Hunter et al., 2019). This finding opened the possibility that activation of the SAN transcriptional program required the blocking of TAK1 phosphorylation targets. To test this, EBs were differentiated in the presence of 5z-7-oxozeanol (OXO). OXO blocks the phosphorylation of TAK1 (Guan et al., 2017; Kobayashi et al., 2018; Liu et al., 2021; Nakajima et al., 2017; Uchida et al., 2017; Wang et al., 2023, 2022; Zhang et al., 2017). Consequently, the phosphorylation of its downstream targets is unidentified (Ishitani et al., 2003; Ishitani et al., 1999; Moriguchi et al., 1997; Ninomiya-Tsuji et al., 1999; Shirakabe et al., 1997). OXO-treated EBs beat faster than untreated EBs and had a similar range of beat rates as our original PGK::TAK1 cell line and the TAK1 inducible EBs (Figs 1 and 6A). OXO treatment also impacted the morphology of cardiac cells at day 21 (Compare Fig. 6B to Fig. 6C) with cells adopting a spindle-like morphology, which is characteristic of SAN cells *in vivo* (Wu et al., 2001). To test whether individual cardiomyocytes activated the SAN transcriptional program, immunocytochemistry was performed to assess Islet1 and Shox2 expression. OXO treatment dramatically increased the percentage of cardiac cells expressing these SAN transcription factors and the percentage of cardiac cells expressing the SAN channel protein HCN4 (Fig. 6C). These data suggest that the SAN transcriptional program can be activated by blocking the phosphorylation of TAK1.

## DISCUSSION

Recent single cell RNA seq data suggest that the SAN is made up of at least five different cell populations including cardiac pacemaker cells (Liang et al., 2021). Pacemaker cells in the SAN are characterized by several molecular and physiological criteria including fast rate of beat, the ability to beat without external activation, expression of Tbx5, Shox2 and HCN4 and the absence of Cx43 expression. Pacemaker cells are also the only population of cardiac cells that expresses the inward funny current (I_f_) (DiFrancesco et al., 1986; Maltsev and Lakatta, 2012). Here, we isolated and analyzed cell lines overexpressing Tbx5 and found that Tbx5 overexpression increased the rate of beating in cardiac cells across a range of doses but did not activate other characteristics of SAN cells.

Tbx5 is initially expressed throughout the cardiac crescent but becomes restricted to the sinoatrial region during linear heart tube formation. Both homozygous and heterozygous loss of Tbx5 causes conduction system defects and loss of SAN structures (Bruneau et al., 2001; Moskowitz et al., 2004), demonstrating that it plays an essential role in the development of the SAN. By contrast, when Tbx5 is ectopically expressed in ventricular tissues, as seen in the Mef2C–/– mouse (Vong et al., 2006), ventricular cells do not convert to an SAN-like identity but rather adopt an atrial fate. Together, these data suggest that overexpression may not be sufficient for cells to adopt SAN fates generally, or cardiac pacemaker fates specifically. Our data appear to agree with this general finding, as EBs overexpressing Tbx5 did not adopt indicators of SAN differentiation other than an increased rate of beating.

Interestingly, in these studies, Tbx5 overexpression resulted in the upregulation of the MAP kinase signaling protein MAP3k7/TAK1. EBs overexpressing TAK1 under the control of a strong mammalian promoter PGK preferentially differentiate cardiac cells to the SAN fate, including expression of the pacemaker current I_f_ (Brown et al., 2017). This suggests that these cells are mostly becoming cardiac pacemaker cells.

We considered two possibilities to explain why TAK1 upregulation downstream of Tbx5 activation did not increase SAN differentiation. First, SAN differentiation by Tbx5 might depend on the dosage of Tbx5. This is unlikely since we found that fast-beating cardiac cells were activated at a particular dosage of DOX, but that increasing dosage had no further impact on beat rate (Fig. 1). This does not eliminate the possibility that TAK1 dosage is important however in these TAK1 expression was upregulated about threefold which is similar to the upregulation observed in the TAK1::PGK cell line (Brown et al., 2017). Alternatively, we considered that SAN differentiation in the PGK::TAK1 line might be an unintended consequence of forced TAK1 overexpression. Several lines of evidence support this idea. First, in the PGK::TAK1 cell line, we noted that while total transcription of TAK1 was increased, that transcription from the endogenous TAK1 promoter was actually repressed (Brown et al., 2017). Indeed we later demonstrated that phosphorylation of known TAK1 targets was repressed during the differentiation of PGK::TAK1 cells (Hunter et al., 2019). This suggests that TAK1 levels are tightly controlled *in vivo*, and that forced overexpression activates mechanisms to downregulate signaling through that pathway. Second, norepinephrine which is known to phosphorylate TAK1 (Akiyama-Uchida et al., 2002), did not activate SAN differentiation in wild-type ES cells (Brown et al., 2017). These data suggest that supraphysiological and unregulatable levels of TAK1 are required to activate SAN differentiation. Although TAK1 has many known downstream targets, the list of potential targets increases steadily. Studies from other groups have also demonstrated that overexpression of TAK1 can inhibit the phosphorylation of downstream targets in a context-dependent fashion (Ajibade et al., 2012; Cheung et al., 2003). Understanding this thoroughly will require studies to determine which of TAK1's known targets (Ishitani et al., 2003; Ishitani et al., 1999; Moriguchi et al., 1997; Ninomiya-Tsuji et al., 1999; Shirakabe et al., 1997) are impacted and whether there are additional as yet unidentified TAK1 phosphorylation targets. Identifying these pathways should lead to improved protocols for SAN differentiation *in vitro*. For example, suramin, which inhibits the phosphorylation of Jnk (Ishitani et al., 2003; Ishitani et al., 1999; Moriguchi et al., 1997; Ninomiya-Tsuji et al., 1999; Shirakabe et al., 1997) enhances SAN differentiation.

The impact of TAK1 on SAN has not been studied *in vivo* because both the original gene trap of TAK1 (Jadrich et al., 2006) and studies knocking it out in the entire heart using a floxed allele (Xie et al., 2006) die before SAN formation is complete.

The current gold standard for differentiating SAN-like cells from human pluripotent cells involves temporal manipulation of the BMP signaling pathway (Protze et al., 2017). This protocol results in the differentiation of a population of cells that are highly enriched for sinoatrial node cells. However, both single-cell patch clamp data (Protze et al., 2017) and single-cell RNA seq data suggest that only a small percentage of these cells represent cardiac pacemaker cells (Liang et al., 2021). To date, this is the only protocol that has been demonstrated to activate a full range of SAN cell types. Activation of cardiac pacemaker cells in this protocol could be due to interaction with the TAK1 signaling pathway. The TAK1-p38 MAPK or TAK1-JNK pathways can be regulated in positive or negative manners by inhibitory SMADs. For example, SMAD7 activates the TAK1-p38 MAPK pathway in human prostate cancer cells (Edlund et al., 2003), whereas Smad6 inhibits TGF-β1-induced activation of this pathway (Jung et al., 2013). In addition, it is known that canonical BMP/SMAD signaling is temporally regulated during cardiac differentiation, with activation required for early cardiac differentiation but repression of those same pathways required for later specification of cardiac cell types (Guzzo et al., 2007).

These studies further elucidate the role intracellular signaling in specifying cardiac cells to the SAN fate. In addition, these data suggest that activation of the SAN transcriptional program may require the repression of TAK1 downstream targets and may represent a simplified protocol for differentiating SAN cells *in vitro*.

## MATERIALS AND METHODS

### Cell culture and embryoid body (EB) differentiation

R1 mouse embryonic stem cells (ATCC) were transduced with aMHC::GFP to track cardiac differentiation (Brown et al., 2010). These cells were subsequently transduced with lentiviruses encoding DOX-inducible-overexpression cassettes for Tbx5 (pTripZ-mTbx5), Tbx3 (pTripZ-mTbx3), Tbx18(pTripZ-mTbx18, Shox2 (pTripZ-mShox2), Islet1 (pTripZ-mIsl1), and MAP3k7/TAK1 (pTripZ-mTAK1). Clonal lines were isolated and individually verified for the expression of genes of interest. MAP3k7/TAK1-overexpressing mESCs was previously described in Brown et al., 2017. These mESCs were maintained in standard growth medium and kept under constant selection with puromycin to maintain cells with continued expression of the pTripz-lentiviral constructs (2 mg/ml). For EB differentiation, these mESCs were passaged off feeder cells (mouse embryonic fibroblasts) and differentiated as EBs using the hanging drop method, as previously described (Brown et al., 2010). On appropriate days, EBs were treated with either DOX diluted in DMSO or DMSO alone. Cell lines are regularly tested by PCR for mycoplasma contamination and tested by PCR for fidelity of insert. Validated cell lines are grown using the Batch Lot system to minimize cross contamination and to prevent variability due to clonal differences. Clonal cell lines will be made upon request to the correspond author.

### Construction of pTripZ-DOX-inducible overexpression vectors

The open reading frames of mouse Tbx5, Tbx3, Tbx18, Shox2, or TAk1 were amplified by PCR and directionally cloned downstream of the TurboRFP reporter using ClaI and MluI restriction sites in the pTripZ vector (Addgene) viral constructs were then validated by sequencing. Lentiviruses were produced using the second-generation lentiviral expression system (Bajpai and Terskikh, 2007). Plasmids will be made available upon request to the corresponding author.

### Real-time quantitative PCR (qRT-PCR)

Approximately 100,000 ES cells were collected for each condition or time point analyzed. Total RNA was extracted with RNeasy Mini Kit, and 200 ng was used for first strand cDNA synthesis using QuantiTect Reverse Transcription Kit. qRT-PCR was performed with SybrGreen Master Mix, using 40 ng template/reaction on a Roche LightCycler 480 Real-Time PCR Instrument, and analyzed with the LightCycler 480 software package. Crossing point data was first adjusted to reflect the efficiency of primer pairs compared to standard curves (based on dilution series over a total dynamic range of 1:1000 or 1:10,000 for positive control cDNAs) and subsequently normalized to GAPDH. Data presented as averages ±standard error (s.e.) of three independent experiments. Further analysis was carried out using GraphPad Prism. Primers used in this study are as follows:


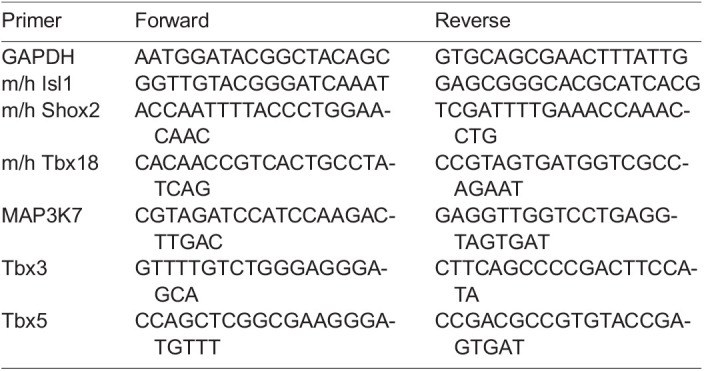


### Flow cytometry

EBs were dissociated with 0.25% Trypsin/EDTA at 37°C for 30 min filter through a 100 µm sieve to remove remaining undissociated cells and debris and resuspended in FACS buffer (PBS plus 1% BSA and ten ng/ml DNAse). Cells were counted using a hemocytometer, checking single-cell suspension simultaneously. Flow Cytometry was performed with The Beckman Coulter MoFlo Astrios EQ cell sorter, and data were analyzed using FlowJo VX software. Cardiac cells were sorted based on the expression of GFP driven by the αMHC promoter, as previously described (Brown et al., 2010).

### Immunocytochemistry

EB dissociation was performed as described above for flow cytometry. Cells were replated onto gelatin-coated four-well chamber slides and allowed to attach for 18-24 h. Slides were fixed in 4% paraformaldehyde for 15 min, gently washed three times for 5 min in phosphate buffered saline (PBS) and blocked with either cytosolic antibody staining buffer (CASB) (PBS, 1% FBS, 0.1% BSA, and 0.1% TritonX-100) or nuclear antibody staining buffer (NASB) (1% FBS, 0.2% BSA, and 0.25% Triton-X-100) for 1 h. Cells were incubated with primary antibodies diluted in blocking buffer overnight. Cells were washed with PBS three times for 5 min and then incubated with Alexa Fluor-labeled secondary antibodies for 1 h at room temperature. Three additional PBS washes were performed with DAPI added to the second wash. Coverslips were added and sealed with acrylic. Unless otherwise stated in the text, images were obtained using a Zeiss AxioImager microscopy. The primary antibodies were used as follow: anti-Cx43 (Sigma-Aldrich, #C6219, 1:1000), anti-CT3 (DSHB, #CT3, 1:250), anti-DsRed (Clontech, #632392, 1:500), anti-GFP (ThermoFisher Scientific, #A11122, 1:500), anti-HCN4 (Sigma-Aldrich, #SAB520035, 1:1000), anti-Shox2 (Abcam, #ab55740, 1:1000), anti-Tbx5 (ThermoFisher Scientific, #42-6500, 1:500), asnti- Islet1 (Abcam, #ab20670, 1:500).

### Bradford assay and western blots

Protein was extracted from different cell lines in RIPA buffer with protease inhibitors and sodium orthovanadate. Total protein was quantified by Bradford assay (Pierce Coomassie Protein Assay Kit) compared to a serial dilution of BSA in a range of 0.064-2 mg/ml. For western blots, 20 μg/protein was loaded per well into prepared SDS-PAGE gels (Invitrogen NuPAGE 12% Bis-Tris gels). Proteins were transferred to PVDF membranes and immunoblotted for visualization with Immobilon western chemiluminescent Horse radish peroxidase (HRP) substrate (Millipore) using a GeneGnome XRQ-chemiluminescence imaging system. Quantification was based on raw peak volume data normalized to the raw peak value for GAPDH. Antibodies used were anti-Tbx5 (ThermoFisher Scientific, #42-6500, 1:500), anit-TAK1 (Sigma-Aldrich, #AB1305414, 1:1000), anti-HCN4 (Invitrogen, MA3-903, 1:1000), HRP conjugated anti-GAPDH (Invitrogen, #MA5-31457, 1:5000) and anti-Cx43 (Sigma-Aldrich, #C6219, 1:1000). Statistical significance was determined by student *t*-test with *P*<0.05 considered significant.

### Beat rate

During EB differentiation, live cell imaging was used to determine the rate of beating for cardiomyocytes (identified based on the expression of αMHC::GFP). The rate was determined by manually counting beats per minute.

### Statistical analysis and data availability

Figures 1 and 2 contain real-time PCR data with stats with statistical analysis performed using proprietary software. Data is presented in the format provided by the Roche software LightCycler software (.ixo files or .csv files for the original run data will be provided upon request to the corresponding author). Other statistical analysis was performed using GraphPad Prism software with statistical type determined by the data in each figure and described in each figure legend. Original metadata will be provided on request.
